# Retraction: Lycopene Inhibits NF-kB-Mediated IL-8 Expression and Changes Redox and PPARγ Signalling in Cigarette Smoke–Stimulated Macrophages

**DOI:** 10.1371/journal.pone.0102411

**Published:** 2014-07-08

**Authors:** The PLOS ONE Editors

The *PLOS ONE* editors retract this publication according to the recommendation received from the Catholic University, Rome.

Upon the publication of the Correction on the article, the *PLOS ONE* editors contacted the Catholic University to note the concerns identified in relation to the presentation of Western blot results from this study. The Catholic University established an investigation commission which has confirmed that several of the figures were inappropriately assembled by the corresponding author Dr. Paola Palozza and advised the retraction of the article.

In line with the institution's recommendation, *PLOS ONE* retracts this publication.
